# Pharmacist gatekeeper interventions for suicide prevention: how evidence from developed countries support their role in low- and middle-income countries

**DOI:** 10.3389/fpsyt.2024.1508621

**Published:** 2025-01-28

**Authors:** Zixiao Zhou, Fahim Mohamed

**Affiliations:** ^1^ Faculty of Medical and Health, The University of Sydney, Sydney, NSW, Australia; ^2^ South Asian Clinical Toxicology Research Collaboration, Faculty of Medicine, University of Peradeniya, Peradeniya, Sri Lanka

**Keywords:** suicide prevention, gatekeeper training, community pharmacist, self-poisoning, LMICs

## Abstract

**Background:**

Approximately 70% of self-poisoning suicides occur in low- and middle-income countries (LMICs).The implementation of pesticide bans has significantly reduced the rate of pesticide self-poisoning in these regions; however, this has been accompanied by a shift toward an increased incidence of pharmaceutical poisoning, highlighting the importance of intervention strategies to prevent pharmaceutical self-poisoning in the future. This report summarizes the existing evidence on community pharmacist gatekeeper interventions aimed at reducing pharmaceutical suicide to discuss their complementary role with pesticide bans in LMICs.

**Methods:**

The literature review identified studies published between April 2014 and April 2024 using multiple keywords related to “suicide,” “intervention,” “pharmacist” and “gatekeeper” in various library databases. Data were extracted into a table for analysis.

**Results:**

Only eight relevant studies were found during the search period, and none quantified the impact of pharmacy gate keeper interventions. Community pharmacists became more confident and willing to intervene after pharmacist gatekeeper training. They demonstrated positive attitudes and improved knowledge and skills in responding to suicidal intent. However, the evidence supporting community pharmacy gatekeeper interventions primarily comes from developed countries. Furthermore, the role of pharmacists in preventing suicide relies on frequent contact between suicidal individuals and pharmacies in developed countries.

**Conclusion:**

Pharmacy gatekeeper interventions can be implemented in LMICs as a complement to pesticide bans, provided they are modified and adapted to suit the specific context of these regions. Further research is essential to tailor and implement successful strategies from developed countries to address the unique challenges faced by LMICs.

## 1 Introduction

According to the World Health Organization, self-poisoning has become one of the most common method of suicide particularly in low- and middle-income countries (LMICs) (1). In 2019, more than 20% of suicides were attributed to self-poisoning (1). Additionally, majority of world’s suicide deaths (77%) occur in LMICs (2), with India and China alone accounting for 42% of the 703,000 annual suicides (1). However, less than 15% of studies on suicide prevention are based in LMICs (3). These facts highlight the importance of learning from the experiences of developed countries to curb self-poisoning prevention in LMICs.

A review of toxicological autopsy data shows that, in developed countries, medically prescribed drugs such as antidepressants are the primary cause of self-poisoning suicides (4). Consequently, these countries have developed suicide prevention through pharmacy regulations, with community pharmacists playing a pivotal role. Pharmacists serve as gatekeepers, regulating access to lethal medicines and act as sentinels by recognizing the signs of individuals at risk of suicide, such as changes in behavior or mood (5). At the level of behavioral prevention, pharmacists can engage with populations at risk and positively influence their mental states to help prevent suicides (6). In addition, pharmacists can also collaborate with other healthcare providers, such as physicians and mental health professionals, or make referrals to appropriate services and resources for individuals at risk of suicide (6).

Due to their critical role in suicide prevention, gatekeeping is an essential skill that supports community pharmacists in managing patients who present with warning signs. It involves recognizing signs of suicide, responding to patients with suicidal thoughts and reassuring them (7). Moreover, training is a key factor shaping the profession of pharmacists. It has an impact on their attitudes, experiences, and preparedness to participate in suicide care (6). Thus, suicide gatekeeper training programs, such as Pharm-SAVES (Signs, Ask, Validate, and Encourage) and QPR (Question, Persuade, and Refer) are designed to help increase suicide prevention rates. These programs can improve the trainees’ knowledge and self-efficacy, build their confidence in detecting signs of suicide and enable them to respond effectively to patients with suicidal thoughts (8).

Although no studies have directly identified a causal relationship between the implementation of community pharmacist gatekeeper interventions and changes in suicide rates, a study of suicide patterns in the United States (US) cited a significant decline in drug-related suicide rates (9). Following the development of the Pharm-SAVES online training program (10), the US suicide rate from drug use dropped by 6% and 7% in 2018 and 2019, respectively (9). In addition, self-poisoning suicide rates have declined since the mid-2000s (9), corresponding to the period when QPR was introduced and began to grow in popularity (11). This trend indicates that pharmacy gatekeeper interventions may be effective.

In LMICs, most suicides by self-poisoning occur through the use of lethal pesticides (4). However, the rate of pesticide self-poisoning in LMICs has declined in recent years following the ban on several highly toxic pesticides (12–14). LMICs are expected to observe trends similar to those in developed countries, with pharmaceutical poisoning becoming the main source of suicide (4). Thus, pharmacy gatekeeper training could emerge as a primary intervention strategy in these resource poor settings to prevent self-poisoning.

In addition to differences in choice of agents for suicide, LMICs differ from developed countries in terms of access to psychological support. Residents of developed countries are accustomed to seeking help from psychiatrists and psychologists. On the contrary, help-seeking behaviors in LMICs often heavily rely on community and family support systems (15). Furthermore, mental health services in developed countries are well-structured to provide a broader psychological support and treatment. In contrast, LMICs often have limited access to mental health services due to lack of medical resources, trained professionals, and healthcare infrastructure. Mental health facilities often unavailable in many rural areas, making it difficult for individuals to access appropriate psychiatric care (16). Therefore, there is a need to review studies on the effectiveness of pharmacy gatekeeper interventions and integrate them to the specific cultural and resource context of developing countries. Integrating these interventions with existing community-based support systems could improve their relevance and effectiveness.

This review aims to explore the effectiveness of pharmacist gatekeeper training interventions implemented in developed countries for reducing self-poisoning suicides. Moreover, it seeks to explore their feasibility for implementation in LMICs. It also evaluates whether pharmacy gatekeeper training programs can emerge as a primary intervention strategy for preventing suicide by drug use.

## 2 Materials and methods

In this review, we analyzed the effectiveness of community pharmacy gatekeeper training as a suicide prevention intervention based on existing evidence. Additionally, we also reviewed other existing gatekeeper interventions to explore feasible self-poisoning prevention interventions appropriate for LMICs.

### 2.1 Rationale behind the study design selection

This article is a semi-systematic literature review, adopting a part of the systematic review checklist from the Preferred Reporting Items for Systematic Reviews and Meta-Analyses (PRISMA) 2020 guidelines (17) to structure its methodological flow. Current research on the topic of “pharmacist gatekeeper interventions” has been conducted by teams of researchers from different disciplines, employing various approaches including both qualitative and quantitative. Thus, the heterogeneity in these studies limits a comprehensive systematic review and meta-analysis.

Semi-systematic evaluations may adopt a more flexible approach to study selection, data extraction, and synthesis. While they follow a structured process similar to that of a systematic review to search and select studies, but do not strictly adhere to all the rigorous methodological criteria of a formal systematic review. Therefore, the semi-systematic method was implemented.

### 2.2 Search strategy

Data were collected from various online libraries including PubMed, ScienceDirect, ClinicalKey, ProQuest, and Cochrane. The databases and search engines used for searching included Medline via Ovid, APA PsycINFO via Ovid, Medline via EBSCOHost, Google Scholar, and the University of Sydney (USYD) Library’s direct on-site search. The initial search used the keyword. “gatekeeper” OR “gatekeeping” OR “gatekeep”, combined with to the term “pharmacist” OR “pharmacy” using the Boolean operator AND.

The initial search was then combined using the operator (AND) with keywords “suicide” OR “self-poisoning”, “prevention” OR “intervention” respectively. Additionally, to identify records related to the implementation of the pharmacist gatekeeper interventions in LMICs, the initial search was further refined using (AND) with keywords “LMICs” OR “low- and middle-income countries” OR “low- and middle-income countries” OR “developed countries”. The search also included more specific terms for common LMIC regions, such as “Middle East”, “Asia”, “Africa”, “South America”, along with more specific country names such as “India”, “China”, “Cambodia”, “Sri Lanka”, “Nepal”, “Pakistan”, “Bangladesh”, etc.

### 2.3 Inclusion criteria

This study adhered to the inclusion criteria outlined in the Joanna Briggs Institute (JBI) Manual for Evidence Synthesis (17). The inclusion criteria include that the article must be in English, published between April 1, 2014, and April 1, 2024, contain an abstract, be available in scientific databases, and evaluate community pharmacist gatekeeper interventions. As several studies reported multiple bans of lethal pesticides before 2014 in many LMICs (18–20), these studies use 2014 as the end of their study periods. We believe that the effects of banning have not been relatively fully recognized and assessed by previous studies before this time point. As a result, recent 10 years window was chosen to minimize the confounding effects of pesticide bans and to include relatively up to date studies.

### 2.4 Definition: low- and middle-income countries

The World Bank Group classifies the world’s economies into four income groups: low-income, lower-middle income, upper-middle income, and high-income. The countries included in each classification are updated on July 1 of each year based on the previous year’s Gross National Income (GNI) per capita (21). The term LMICs (low- and middle-income countries) includes all low-income, lower-middle-income and upper-middle-income countries.

### 2.5 Definition: gatekeeper intervention

Suicide prevention “gatekeepers” are people who can contact individuals at high risk of suicide and have the opportunity to prevent suicides. At the societal level, gatekeeper interventions consist of educating individuals with the knowledge, skills and confidence to identify and support those at risk (8). In community pharmacy, gatekeeper interventions can also refer to specific proactive actions taken by community pharmacy staff, including identifying, communicating with, and assisting patients who present with warning signs of suicide (10).

### 2.6 Data analysis

The screening of titles and abstracts was carried out by ZZ during the literature search and data analysis. Data were entered into a custom data extraction sheet (**Table 1**). The main information collected include participant characteristics, key concepts, primary results or conclusive outcomes, and the methodology and limitations used to evaluate the quality of the study.

**Table 1:** Data extraction sheet: Summary of key study characteristics.

Authors/publication year	Study design and population	Gatekeeper intervention and indicators of efficacy	Key findings	Limitations
Nagashima et al. (2024) (23)	Survey and Logistic Analysis 162 pharmacists and 136 registered sellers (Japan)	No specific training programs. Education and training about knowledge of prescription and drugs are involved. Key indicators besides demographic information from the survey include experience with overdose situations, knowledge of drugs, countermeasures at the workplace, participation in educational activities and willingness to participate in future training.	The odds ratio for the willingness to participate in study sessions and workshops about overdose and likelihood of intervening drug overdose was found to be 3.5 in registered sellers, indicating a strong association. Gatekeeper training plays an important role in providing pharmacists and registered sellers with countermeasures to prevent overdose poisoning from drugs.	Self-selection bias. Poor generalization to other countries or regions with different contexts.
Stover et al. (2023a) (24)	Scoping Review Community pharmacists and student pharmacists, from seven published research articles	Reviewed pharmacists gatekeeper training programs include Mental Health First Aid (MHFA), Question, Persuade, and Refer (QPR), Optimizing Suicide Prevention Programs and Their Implementation in Europe (OSPI-Europe), Social Worker Pharmacist Collaborative Didactic Lecture, and Suicide Prevention for Pharmacy Professionals. The main indicators are training outcomes included changes in communication skills and ability to identify suicide warning signs and referral resources.	Most of the training (86%) facilitated the practice of verbal and behavioral skills through live interactions or role-plays. All programs improved participants’ ability to recognize suicide warning signs and refer to resources. Approximately half of the programs (57%) demonstrated improvements in participants’ knowledge while fewer (29%) showed improvements in communication skills.	All articles were reviewed and compiled by a single researcher potentially introducing bias.
Stover et al. (2023b) (25)	Interview A community pharmacy stakeholder panel of nine community pharmacy staff, and a veteran stakeholder panel of 10 veterans from all branches, service periods, races, and genders, including both officers and enlisted members (United States)	Pharm-SAVES (Recognize the warning Signs, Ask if someone is considering A suicide,Validate feelings to encourage open communication, Expedite referral to resources, Set a reminder to follow up) is a brief, online suicide prevention gatekeeper training program specifically designed for community pharmacy staff. No specific indicators applied for testing efficacy.	Pharm-SAVES was co-designed by pharmacy and veteran stakeholders. The combination of targeted training, stakeholder involvement, interactive learning, and a focus on communication makes Pharm-SAVES an effective tool for enhancing the role of pharmacy staff in suicide prevention efforts.	This interview primarily focuses on the development of gatekeeper training modules and provides indirect evidence only.
Pothireddy et al. (2022) (26)	Longitudinal Observational Study 139 student pharmacists, 63% in the second year, 37% in the third year; 16% expected to work in the community. The demographic included 21% Asian, 7% Black or African, 64% White, and 5% identified as Other. (United States)	SAVE (Signs, Ask, Validate, Encourage and Expedite Referral) is the previous version of Pharm-SAVES, a gatekeeper training program developed by the Veteran Administration (VA). The main indicators are students’ ability to recognize warning signs, behavioral changes as responses to hypothetical scenarios, engagement with the training processes and changes in students’ knowledge and confidence regarding suicide prevention.	After learning SAVE, students gain confidence and knowledge in identifying and managing suicide warning and signs in facilitating referrals. The percentage of students who validated the individual’s feelings increased from 41% to 82%. Furthermore,86% of students believe it can be applied in practice.	Self-selection bias. Differences in class length and schedules among schools may have caused differences in learning. Generalizability is limited because only second- and third-year students were assessed.
Hawgood et al. (2021) (7)	Longitudinal Observational Study 299 individuals from diverse professional backgrounds: 15% male, 85% female. Most participants were aged above 35 years with 35% having never worked in suicide prevention and 60% health workers. (Australia)	Wesley LifeForce is a full day suicide prevention training program. A 6-month follow-up evaluation of perceived capability, attitudes toward suicide prevention and reluctance to intervene.	Wesley LifeForce improves participants’ confidence, proactive willingness, positive attitude, and knowledge related to intervening in suicide prevention efforts, with lasting results.	Self-selection bias. Lack of a control group to compare the effects of training. High attrition rates.
Carpenter et al. (2021) (10)	Semi-structured interview 17 pharmacists: 5.9% male, 94.1% female. The majority were in the 35–54 age group. The demographics were as follows: 70.6% White, 5.9% Black or African American, 5.9% Asian, and 17.6% Other (United States)	SAVE. Main indicators are confidence in communication, referral rates, patient outcomes and feedback from participants.	Participants emphasized the importance and significance of SAVES gatekeeper training needs and expressed the importance of knowing suicide statistics, warning signs, risk factors, referral procedures, and effective communication strategies with at-risk patients.	Self-selection bias. The sample was skewed toward more females, White, younger, and more likely to work at independent pharmacies and interested in suicide prevention than the general population.
Murphy et al. (2018) (5)	Survey and Thematic Analysis 176 pharmacy staff, 69% male, 31% female. Mean age 41, range 22–75 years; 57% from Canada, 43% from Australia	Training with screening tool Patient Health Questionnaire (PHQ), MHFA and OSPI-Europe. Participants’ self-reported confidence, changes in perceptions regarding suicide and their willingness to engage with individuals at risk are measured.	Gatekeeper training can improve pharmacists’ confidence in addressing suicide risk, enhance their knowledge of available resources, and improve their communication skills when dealing with individuals in crisis.	Reliance on free-text responses from an online survey without the opportunity for further insights from interviews or focus groups.
Painter et al. (2018) (11)	Survey and Pretest-posttest evaluation 103 pharmacists, 41% male, 59% female; mean age 43 ± 16 range 22–74; 40% Asian and 46% White; 45% of respondents reported knowing someone who died by suicide. (United States)	QPR, a training program which is designed to provide individuals with the basic skills necessary to recognize a crisis and the warning signs of suicide, enabling them to refer someone to help. Participants were asked about their confidence in identifying signs of suicide, listening without judgment, responding appropriately to patients with suicidal thoughts and changes in attitude.	QPR gatekeeper training enabled 70%–90% of participants to update their knowledge following the training and feel more confident to intervene with patients at risk of suicide.	Self-selection bias. Failure to assess sustainable effects following a short training period.

In the absence of records establishing a statistical relationship between the implementation of pharmacist gatekeeper training programs and changes in suicide rates, qualitative indicators (attitude, knowledge, behaviors, etc.) were employed to assess the efficacy of these interventions in the countries where they were implemented. In this study, we focused on the narrative outcomes of the analyzed studies, for example, subjects’ perceptions of intervention strategies and changes in attitudes before and after their participation.

### 2.7 Ethics statement

According to ethics approval guidelines (22), the ethical review process is mandatory for designing, organizing, or conducting research that directly or indirectly involves patients. This literature review involved only the synthesis and analysis of published studies; no new research involving human subjects was conducted. Therefore, formal ethical approval was not sought. All sources of information are publicly available, and references were properly cited to respect the intellectual property rights of the original authors.

## 3 Results

**Figure 1:**
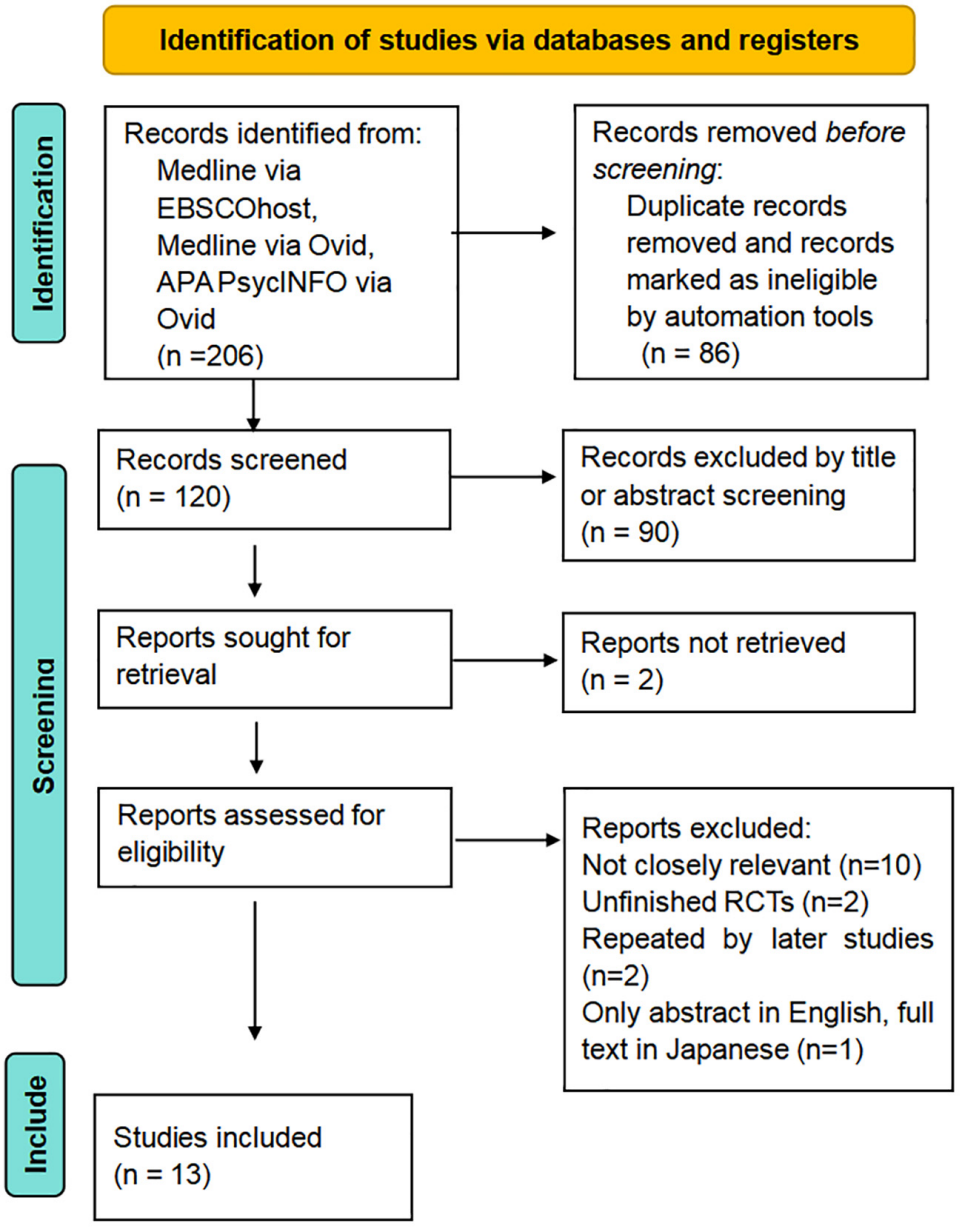
Prisma flow diagram of selection of articles on pharmacy interventions.

Two hundred and six studies were identified through database searches (**Figure 1**). All 206 search results were integrated using the command ‘search with OR’ to remove duplicates, and some results were automatically excluded by database search engines after setting inclusion criteria. Subsequently, 120 studies were screened, and 28 of them were selected to assess their eligibility. Two sources could not be retrieved due to a lack of free online access, while 15 studies were excluded for reasons of inaccessibility, low relevance, and other factors. Ultimately, 8 studies were selected to summarize the pharmacy gatekeeper interventions (5, 7, 10, 11, 23–26), (**Table 1**). Five relevant studies were selected and evaluated to discuss the current profile of self-poisoning in LMICs (27–31).

Of the eight studies summarisedin **Table 1**, five were from the US, three from Australia, and one from Japan (two studies spanned two countries). Five studies recorded the gender and age, while three documented the ethnicity. Most participants were aged between 30 and 50 years. The participants included qualified pharmacists, student pharmacists, pharmacy technicians, registered sellers, pharmacy owners, and other pharmacy-related staff.

While current evidence on the effectiveness of pharmacist training programs originated primarily from developed countries, a significant gap remains in the literature with respect to adaptation and implementation of pharmacist gatekeeper training in the LMIC context. No relevant results found when combining the topic of pharmacist gatekeeper with any keywords referring to LMICs. However, we retained five studies that, although not directly addressing pharmacy gatekeeper interventions, examine drug-related suicide or explored appropriate methods of suicide prevention strategies in LMICs (27–31).

Pharmacy gatekeeper intervention strategies were consistently perceived as effective by the majority (86%) of participants across eight studies (24, 26). Training programs such as QPR, SAVE, Pharm-SAVES, and Wesley LifeForce enhanced participants’ ability to recognize warning signs, communicate effectively, and refer at-risk individuals (**Table 1**). For instance, SAVE increased validation of individuals’ feelings from 41% to 82% (26), and QPR achieved 70% to 90% confidence improvements among participants (11). Indicators for measuring the efficacy of interventions are mainly based on pharmacists’ subjective feelings such as changes in attitudes, increased self-confidence, and readiness to respond to suicidal intent. Other relatively objective ability indicators included expanded knowledge and improving pharmacists’ skills.

Only Nagashima et al. emphasized that pharmacists play a gatekeeper role in drug overdose situations, highlighting the need for both theoretical and practical education regarding lethal doses of pharmaceutical drugs and dangerous drug interactions (23). In other literature, the rationale for pharmacist gatekeeper training is not centered on withholding access to medicines but rather on their interactions with individuals at risk of suicide, as pharmacists are the most accessible health professionals to the public (24).

## 4 Discussion

### 4.1 Implications of the evidence for pharmacist gatekeeper interventions

Pharmacists did not play a role as a barrier to prevent pharmaceutical overdoses; rather, they assume on a role resembling that of a psychiatrist working in a pharmacy. It is noted that 80% of suicides in the US had contact with a healthcare provider in the year prior to death (30). Although we were unable to confirm whether such contacts were made to seek support for psychological problems or for other health problems, this data at least proves that there is frequent and close contact between health workers and individuals at-risk in developed countries, which presents the opportunity for prevention. This finding also suggests that the effectiveness of pharmacist gatekeeper interventions is highly dependent on the context of developed countries, where pharmacists have close contact individuals with suicidal risks. In LMICs, however, individuals at risk of suicide often do not receive adequate mental health support from pharmacy or healthcare system (15). Despite the broader role pharmacists have to play by positioning themselves as champions in suicide prevention interventions, implementing such interventions through community pharmacies may be limited in some rural and suburban areas of LMICs, where pharmacies are not widely available (31). Therefore, recommendations for applying the pharmacist gatekeeper training in LMICs must consider unique context and the current trends in self-poisoning in these countries.

Furthermore, although studies have demonstrated the efficacy of pharmacist gatekeeper interventions, “efficacy” reflects an increase in pharmacists’ ability to prevent patient suicides as a result of training, rather than a direct reduction in the number of suicides attributable to this strategy. The direct statistical effect of all gatekeeper keeper training programs on reducing suicide rates, however, remain (32). At the same time, many confounding factors, such as the impact of shifts in the economic environment, likely influence overall suicide rates (3). Thus, even in the context of studies conducted in developed countries, health economics data, such as the cost-effectiveness of pharmacy gatekeeper interventions, remain difficult to measure (33). Nonetheless, pharmacists still have a pivotal role to play in suicide prevention interventions.

### 4.2 The current role of pesticides bans in LMICs

For most countries globally, the most effective suicide prevention strategy involves prohibiting the use of lethal methods at the legislative level (27). Among all these approaches, restricting pesticides is generally considered the optimal option for LMICs. Banning highly hazardous pesticides could reduce suicide deaths by approximately 28,000 per year, at a cost of only 0.007 US dollars per capita annually (34). Current evidence indicates that safe storage methods were ineffective on the incidence of pesticide self-poisoning. In contrast, banning of highly toxic pesticides resulted in reduction in suicides without impacting agricultural output (35).

Due to the recent successful implementation of bans on lethal pesticides in some LMICs (12), researchers have predicted that the primary method of self-poisoning will shift from the use of lethal pesticides to medication overdoses, thereby highlighting the importance of community pharmacies. However, pesticide bans have not completely blocked access to toxic pesticides due to strong resistance from the industry (13, 35). Highly toxic pesticides, such as organophosphates and carbamates, rather than pharmaceuticals, remain the primary method of self-poisoning in LMICs (4). Most victims of poisoning used pesticides because they are not only more lethal but also more widely available, often stored in most households in both urban and rural areas (31). However, emerging trends in LMICs indicate a shift towards pharmaceutical self-poisoning as a result of restricted access to pesticides, highlighting a changing landscape of suicide methods that requires targeted interventions. At this stage, pharmacy gatekeeper interventions should serve as a complement pesticide bans rather than replacing them as the primary focus of suicide prevention intervention in developing countries.

### 4.3 Other gatekeeper interventions for suicide prevention in LMICs

Although LMICs do not have pharmacy gatekeeper interventions equivalent to those in developed countries, other existing gatekeeper interventions in developing countries may be able to serve as a reference for the localization of pharmacy gatekeeper interventions.

The school gatekeeper interventions in China are school education strategies aim to equip teachers, school staff, parents and peers with the skills to recognize the warning signs of students at risk of suicide, based on the idea that recognising suicide risk is key to suicide prevention (36). Because Chinese student population comes into contact with schools on a daily basis, school gatekeepers have an inherent advantage in preventing youth suicide, being able to recognize and deal with suicide risk signals at the first opportunity (37). This is similar to the advantage that pharmacies in developed countries of having frequent access to people at risk of suicide, which suggests that the application of pharmacy gatekeeper interventions in LMICs should first ensure that pharmacy services are able to radiate to living areas of targeted population.

In rural areas, pesticide sellers, rather than community pharmacy staff, often serve as gatekeepers in blocking access to chemical suicide (31, 38), thus a gatekeeper training intervention tailored for pesticide vendors may be applicable in these regions. Trained suppliers can serve as gatekeepers by limiting access to pesticides and by identifying and responding to high-risk individuals among customers (29). The two vendor-based interventions for restricting pesticide sales include selling pesticides only to farmers with identity cards or to customers with pesticide “prescriptions,” and selling low-toxicity products while providing counseling and asking customers to return the next day (28, 39). This is a combination of the pharmacy gatekeeper model and pesticide restriction policies. What we can learn from this is that the “pharmacist gatekeeper” who block lethal drugs do not necessarily have to be pharmacy staff, and that intervention strategies can also consider the channels through where drugs are actually sold in LMICs.

### 4.4 Limitations and strength

This literature review is semi-systematic due to its more flexible structure has some advantages of systematic reviews such as methodological rigor and transparency (40, 41). However, this study is prone to source selection bias and has limited comprehensiveness, as most studies are arbitrarily selected during the process. In addition, the results of studies evaluating the effectiveness of gatekeeper interventions have tend to rely on subjective indicators of attitude, knowledge, and confidence. As a narrative outcome, the efficacy observed is likely influenced by factors such as the placebo effect, measurement errors, and the social dynamics of attending training with peers rather than the course content itself, which can increase participants’ self-confidence. These confounding factors need to be considered when interpreting the results.

This literature review summarizes existing literature on pharmacy gatekeeper intervention strategies worldwide, providing a theoretical framework and preliminary background for future research in drug suicide prevention area. It explores the key role of pharmacists in community suicide prevention interventions and compares the requirements for suicide interventions in developed and developing countries. This study also provides key insights into how community pharmacy gatekeeper interventions can be appropriately modified to achieve effective and feasible outcomes in LMICs.

## 5 Conclusion

Pharmacist gatekeeper training programs offer a proactive approach and are effective in enhancing confidence, willingness, positive attitudes, knowledge, and readiness of pharmacists to identify and assist patients at risk of suicide. The value of pharmacists as suicide gatekeepers lies not only in controlling access to pharmaceuticals, but in their frequent interaction with individuals who may be at risk. Therefore, tailoring gatekeeper training programs to meet these specific needs of LMICs, rather than directly transferring models from high-income countries, is essential. However, significant gaps remain in quantitative research on the efficacy of gatekeeper training programs in LMICs where pharmacist led gatekeeper interventions studies are scarce. At this stage, there is a pressing need for holistic suicide prevention strategies in LMICs, combining effective measures such as pesticide bans with adaptable pharmacist gatekeeper training programs tailored to the local social context. Effective suicide prevention in LMICs requires integrating gatekeeper training and pesticide bans, conducting pilot studies on pharmacist interventions, and addressing broader social and systemic causes.
